# Multi-Dimensional Revealing the Influence Mechanism of the δ Phase on the Tensile Fracture Behavior of a Nickel-Based Superalloy on the Mesoscopic Scale

**DOI:** 10.3390/ma15020610

**Published:** 2022-01-14

**Authors:** Qiang Zhu, Linfu Zhang, Chuanjie Wang, Gang Chen, Heyong Qin, Peng Zhang

**Affiliations:** 1 School of Materials Science and Engineering, Harbin Institute of Technology, Weihai 264209, China; zhuqiang@hit.edu.cn (Q.Z.); 21B909090@stu.hit.edu.cn (L.Z.); cjwang@hitwh.edu.cn (C.W.); cg@hitwh.edu.cn (G.C.); 2 Central Iron & Steel Research Institute, Beijing 100081, China; qinheyong@126.com

**Keywords:** nickel-based superalloy, strain evolution, fracture mechanism, δ phase

## Abstract

As the key materials of aircraft engines, nickel-based superalloys have excellent comprehensive properties. Mircotensile experiments were carried out based on in situ digital image correlation (DIC) and in situ synchrotron radiation (SR) technique. The effects of the δ phase on the grain orientation, surface roughening, and strain localization were investigated. The results showed that the average kernel average misorientation (KAM) value of the fractured specimens increased significantly compared with that of the heat-treated specimens. The surface roughness decreased with an increasing volume fraction of the δ phase. The strain localization of specimens increased with the increasing ageing time. The size and volume fraction of voids gradually increased with the increase in plastic strain. Some small voids expanded into large voids with a complex morphology during micro-tensile deformation. The needle-like δ phase near the fracture broke into short rods, while the minor spherical δ phase did not break. The rod-like and needle-like δ phases provided channels for the propagation of the microcrack, and the accumulation of the microcrack eventually led to the fracture of specimens.

## 1. Introduction

The geometry or feature size of micro-parts is below the millimeter scale, thus their forming belongs to the category of mesoscale plastic deformation. Therefore, it is challenging to form micro-parts with high precision, high quality, low cost, and high reliability [1]. Plastic microforming technology has won the favor of scholars because of its high volume, high productivity, high precision, and no pollution, and has become a key manufacturing technology of micro-parts [2,3,4]. With the unstoppable trend of product miniaturization in many fields such as aerospace, nuclear industry, weaponry, and energy, some essential micro-parts applied in these fields must be able to hand the high temperature, high pressure, special media corrosion, and other harsh environments. Nickel-based superalloys have become critical materials thanks to their excellent high-temperature strength, high oxidation and corrosion resistance, and good fatigue property [5,6,7,8,9].

The mechanical properties of nickel-based superalloys are closely related to the microstructure. Nickel-based superalloys normally exhibit three intermetallic precipitation phases, that is, (i) γ′ with a composition Ni_3_(Al,Ti) and a cubic (L1_2_) crystal structure, (ii) γʺ with a composition Ni_3_Nb and a bct (D0_22_) crystal structure, and (iii) δ with a composition Ni_3_Nb and an orthorhombic (D0a) crystal structure [10]. The δ phase precipitates between about 780 °C and 980 °C. Its precipitation rate is highest around 900 °C. The δ phase normally nucleates and precipitates at grain boundaries followed by the growth of thin plates extending into the grains. The nucleation of the δ phase can also occur intragranularly in the presence of the γ″ phase. The γ″ phase is susceptible to forming an orthorhombic δ phase in the overaged condition or long service. The γ″ and δ phases are Nb-based intermetallics, where the γ″ phase is a metastable phase and the δ phase is the equilibrium one. As a result, the formation of the δ phase occurs at the expense of the γ″ phase, which is associated with the decreased precipitation hardening effect by depletion of the coherent γ″ precipitates [11].

Many studies have been carried out on the macroscopic scale to uncover the influence of the δ phase on the mechanical properties, fracture behavior, and mechanism of nickel-based superalloys [12,13,14,15,16,17]. The δ phase has some beneficial effects. Moderate fractions can effectively limit grain growth during solution treatments [14,15] and the δ phase with an appropriate morphology at the grain boundary has been shown to provide resistance to grain boundary creep fracture [16]. Wu et al. [17] demonstrated that nickel-based superalloy exhibited a certain degree of fatigue resistance when the distribution of the δ phase was not parallel to the loading direction. The granular δ phase distributed at grain boundaries firmly pin grain growth, thus improving the fatigue resistance and stress rupture properties [18]. However, the formation of extensive amounts of the δ phase during service leads to severe degradation of properties and is to be avoided. Lin et al. [19] found that the fracture time and breaking elongation of nickel-based superalloy increased and then decreased with the increasing volume fraction of the initial δ phase. Additionally, the fracture mode changed from typical intergranular fracture to mixed fracture of intergranular and cleavage fracture with the increasing volume of the initial δ phase. Li et al. [20] found that the precipitation of the δ phase improved the tensile strength, so the deformation resistance of the nickel-based superalloy was elevated. However, the plasticity of the nickel-based superalloy exhibited a rise followed by a fall. The long needle-like δ phase was pulled off into short rods along the tensile direction. Ning et al. [21] reported that, while the plasticity of the rod-shaped δ is good, the needle-like δ contributes to increasing strength and reducing ductility.

The current published works about the effect of the δ phase on the fracture behavior of nickel-based superalloys are mainly focused on the macro-scale tensile deformation. On the mesoscopic scale, there are few reports about the influence of the δ phase on the tensile fracture behavior of nickel-based superalloy. Additionally, the current characterization methods to study the effect of δ on the fracture of nickel-based superalloys mainly use two-dimensional (2D) SEM and TEM. In this paper, the effect of the δ phase on the fracture behavior of nickel-based superalloys was investigated on the mesoscopic scale using 2D and 3D characterization methods, which included electron backscatter diffraction (EBSD), confocal laser scanning microscope (CLSM), scanning electron microscope (SEM), in situ DIC, and in situ SR techniques. The fracture mechanism was revealed through studying the effects of the δ phase on grain orientation, surface roughing, and strain localization during microtensile process. The results of this paper can provide theoretical guidance for adjusting the δ phase to improve the service life and reliability of nickel-based superalloys in actual service environments.

## 2. Materials and Methods

The cold-rolled GH4169 nickel-based superalloy sheet of the Central Iron & Steel Research Institute was selected as the experimental material. The thickness of the GH4169 nickel-based superalloy sheet is 200 μm. The tensile strength and the elongation of the as-received material are 850 MPa and 43%, respectively. The chemical composition of the base metal was analyzed by the X-ray diffraction (XRD) technique, which is shown in Table 1.

**Figure 1:**
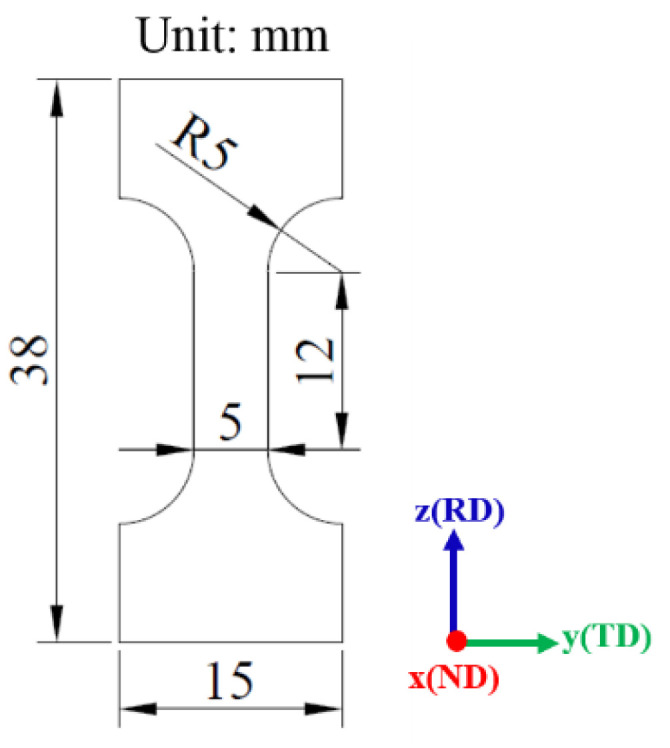
Schematic diagram of the tensile specimen.

Figure 1 shows the schematic diagram of the dog bone shape tensile specimen. The tensile specimens with the gauge width of 5 mm and gauge length of 12 mm were prepared along the rolling direction (RD) using electrical discharge machining (EDM). The cutting edges of the specimens were polished with 2000 grit SiC paper to reduce the impact of EDM on the performance of the specimens. Then, the tensile specimens were cleaned with an alcohol solution in an ultrasonic cleaner. In order to prevent the oxidation of specimens during heat treatment, which could affect the subsequent analysis of mechanical properties and fracture behavior, the vacuum heat treatment method was used. The solution temperature was held at 1100 °C, and water quenching (WQ) was selected so that it could maintain the high temperature microstructure. The precipitation temperature range of δ phase is 780–980 °C, and 900 °C is its precipitation peak temperature. At 900 °C, a large amount of δ phases with different particle sizes and morphologies can be precipitated in a short aging time. In order to better study the influence of the morphology and volume fraction of the δ phase on the tensile fracture, the aging temperature of 960 °C is chosen in this study. The solution-treated specimens were aged at 960 °C for 2 h, 12 h, and 24 h, then WQ was chosen as the cooling method.

In situ tensile experiments based on the DIC technique with a strain rate of 0.001 s^−1^ were carried out on a universal testing machine (Instron5967, INSTRON, Boston, MA, USA) to reveal the local strain evolution rules during plastic deformation. The surface of the specimens was sprayed with speckle to obtain an accurate strain field. First, a thin layer of white paint was sprayed on the specimen surface. Then, black paint was uniformly sprayed on top of the white paint, and it should be ensured that the black paint is randomly and diffusely distributed on the specimen surface. During the in situ tensile experiments, the photos were collected in real time from the initial undeformed to the final fracture at each specimen gauge to ensure that the strain evolution behavior of the microtensile process could be accurately analyzed. Five replicate experiments were conducted for each different microstructure specimen to improve the accuracy of the test.

Based on the SR technique, an in situ loading device was built to realize the three-dimensional characterization of void nucleation and expansion, and to reveal the influence mechanism of microstructure on void and crack nucleation and expansion of GH4169 nickel-based superalloy sheet. In situ microtensile tests were carried out at the BL13W1 beamline of the Shanghai Synchrotron Radiation Facility (SSRF, Shanghai, China). The spatial resolution of the detector was 0.65 μm, which is the nominal isotropic resolution of the 3D reconstructed images. A total of 1000 images were taken with a beam energy of 34 keV over the gauge length of each tensile specimen and over 180°, with a 250 ms exposure time for each image. The quantitative statistics of void defects were obtained using PITRE and Avizo 9.0 software.

In order to reveal the influence of the δ phase on the micro-orientation evolution behavior during plastic deformation, the grain boundary orientation was characterized by EBSD (EDAX, Mahwah, NJ, USA). The specimens across the rolled direction–transverse direction (RD–TD) section were characterized with an accelerating voltage of 20 kV, a working distance (WD) of 15 mm, a scanning step size of 0.5 μm, a binning of 4 × 4, and a 1.4 megapixel resolution of 1392 (H) × 1040 (V). In order to observe and analyze the δ phase morphology and the fracture morphology of the specimens, SEM (ZEISS, Oberkochen, Germany) was chosen. In order to investigate the influence of δ phase on the flow behavior during microtensile deformation of GH4169 nickel-based superalloy, the surface roughness was characterized by CLSM (OLS3000, OLYMPUS, Tokyo, Japan) with a scanning step of 0.3 μm and an observation area of 640 μm × 640 μm on the rolling direction (RD)–thickness direction (TD) plane.

## 3. Results and Discussion

### 3.1. Microstructure Evolution

**Figure 2:**
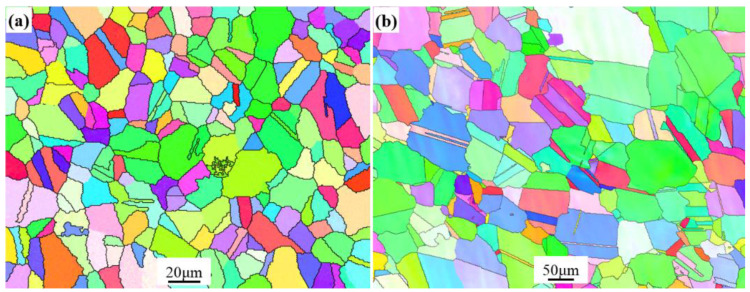
Grain maps of the alloy. (**a**) Original specimen; (**b**) solution-treated specimen.

**Figure 3:**
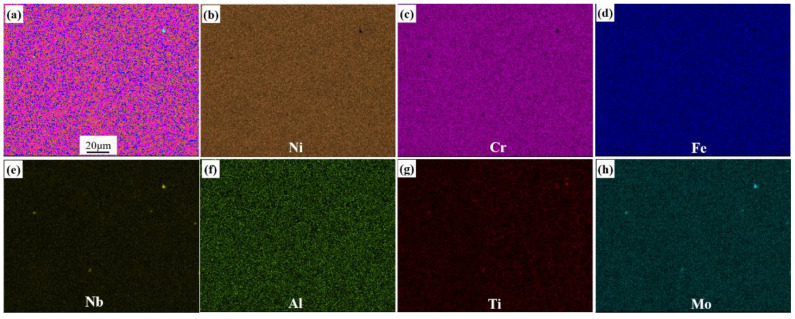
Area scanning results of solution elements for the specimen-treated specimen. (**a**) Distribution of all elements; (**b**) Ni distribution; (**c**) Cr distribution; (**d**) Fe distribution; (**e**) Nb distribution; (**f**) Al distribution; (**g**) Ti distribution; (**h**) Mo distribution.

The metallographic structure of the original GH4169 nickel-based superalloy sheet is shown in Figure 2a. To calculate grain size, edge grains were included in the analysis and twins were not included in the analysis. Additionally, the grain tolerance angle value of 5 was used to define the grains. The equivalent diameter was used for the grain size calculation. It can be seen that the grain size is relatively small, about 12.4 ± 2.2 μm. The GH4169 nickel-based superalloy sheet is annealed during cold rolling to reduce work hardening. Thus, the microstructure of original specimen contains some δ and γ″/γ′ phases. In order to eliminate the texture, δ, and γ″/γ′ phases in the original microstructure, the original GH4169 nickel-based superalloy sheet was subjected to high temperature solution treatment. The metallographic structure after solution treatment is shown in Figure 2b. It can be found that the metallographic structure consists of equiaxed crystals with a grain size of 69.8 ± 3.5 μm. A certain number of twins appear in the metallographic structure, and the grain boundaries are relatively straight. As the solution temperature is higher than the complete dissolution temperature of the δ phase, no δ phase exists in the microstructure after solution treatment. The area scanning results of elements are displayed in Figure 3. It can be observed that the major solid solution elements of Cr, Fe, Nb, Al, Ti, and Mo are homogeneously distributed in the matrix γ phase.

**Figure 4:**
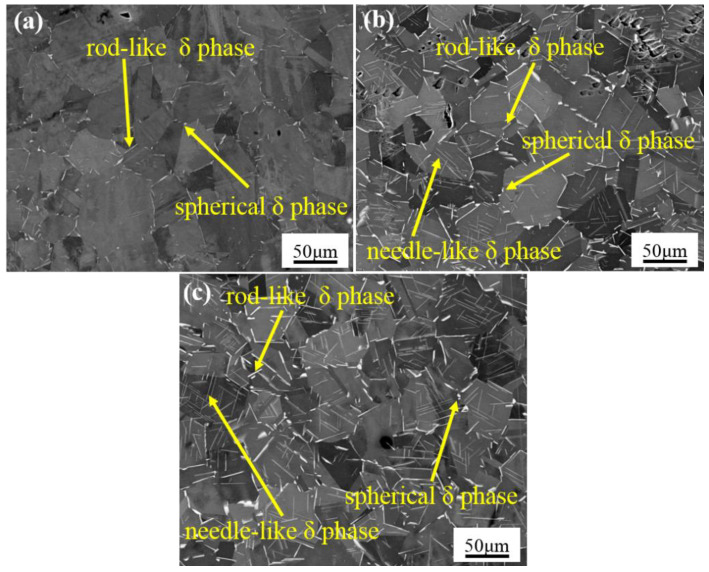
Morphologies of the δ phase after aging treatment for different times. (**a**) 2 h; (**b**) 12 h; (**c**) 24 h.

Morphologies of the δ phase after aging treatment are shown in Figure 4. After aging treatment for 2 h, the spherical and rod-like δ phases precipitated at the grain boundaries, as shown in Figure 4a. After aging treatment for 12 h, the size and volume fraction of the δ phase increased significantly, as shown in Figure 4b. Additionally, some spherical and rod-like δ phases distributed at the grain boundaries evolved into needle-like δ phases. After aging treatment for 24 h, the needle-like δ phases appeared parallel with each other and many spherical δ phases with small size precipitated in the grain, as shown in Figure 4c. As the morphology evolved from the spherical δ phase and rod-like δ phase to the needle-like δ phase, the long-axis size of the δ phase changed from a few microns to tens of microns. The volume fractions of the δ phase after aging treatment for 2 h, 12 h, and 24 h were 1.17 ± 0.12%, 3.21 ± 0.22%, and 4.05 ± 0.18%, respectively.

The precipitation mechanism of the δ phase is controlled by the diffusion of Nb element. The alloy elements are easy to diffuse and aggregate at the grain boundaries or twin boundaries with a higher concentration or free energy. As the aging time increases, the diffusion rate of Nb atoms increases gradually. Therefore, the spherical and rod-like δ phase gradually evolved into the needle-like δ phase. Thus, the morphology of the δ phase can be controlled by adjusting the aging time.

### 3.2. Grain Orientation Evolution

**Figure 5:**
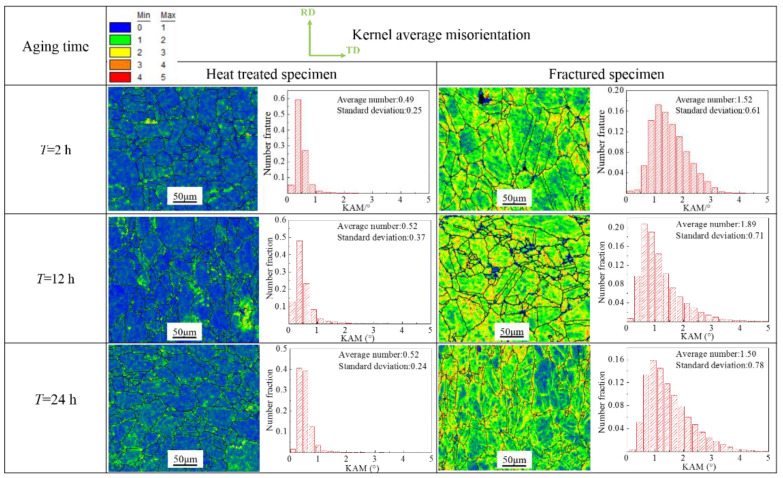
Engineering stress–engineering strain curves of the alloy.

Plastic deformation can be characterized by the local micro-orientation in the material [22]. In order to reveal the influence of the δ phase on the micro-orientation evolution behavior during plastic deformation, the kernel average misorientation (KAM) of the heat-treated specimens and fracture specimens is shown in Figure 5. The KAM values of the heat-treated specimens are low, and the KAM values in the local area are inhomogeneous. The KAM values near the grain boundaries and twin boundaries of the heat treated specimens are relatively higher, which is mainly related to the particle size and distribution of the δ phase. The micron-scale δ phase was preferably generated at the grain boundaries as a spherical or rod-like shape. It would gradually evolve into a needle-like shape and expand into the grain with the increase in aging time. Comparing the KAM distribution curves of heat-treated and fractured specimens, it can be seen that the average KAM values of fractured specimens are considerably higher than those of heat-treated specimens. Additionally, the δ phase impeded the movement of dislocations during the tensile deformation. The stress concentration caused by the accumulation of dislocations near the precipitated phase could not be released in time, which easily produced local strain concentration. Because the δ phase mainly precipitated at the grain boundary, the KAM value at the local grain boundary displayed a greater increase on the KAM graph.

### 3.3. Surface Roughening Behavior

As plastic deformation occurs in the polycrystalline materials, the surface roughening phenomenon inevitably emerges, which affects the subsequent plastic deformation behavior and the formability of the materials. Therefore, surface roughening is one of the crucial problems in the forming process. On the mesoscopic scale, plastic deformation and ductile fracture are very different from that on the macroscopic scale. The surface roughness evolution is mainly influenced by microstructure and strain distribution.

**Figure 6:**
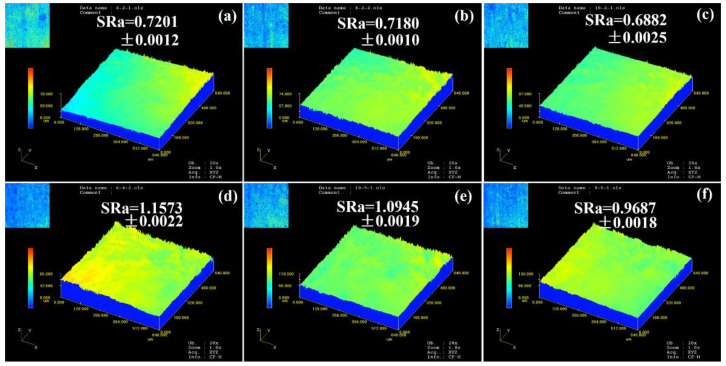
Surface roughness of the deformed specimens. (**a**) T = 2 h, ε = 0.1; (**b**) T = 12 h, ε = 0.1; (**c**) T = 24 h, ε = 0.1; (**d**) T = 2 h, ε = 0.4; (**e**) T = 12 h, ε = 0.4; (**f**) T = 24 h, ε = 0.4.

The macroscopic scale deformation mainly focuses on the “average” result and does not consider the deformation differences of internal and surface grains. The homogeneous deformation on the macroscopic scale will become heterogeneous on the mesoscopic scale owing to the different orientations of the adjacent grains. The surface roughening behavior is closely related to the deformation behavior of individual grains on the mesoscopic scale. The strain in each grain depends on its crystallographic orientation with respect to the applied stress during plastic deformation [23]. Therefore, different orientations between adjacent grains can lead to free surface roughening on the mesoscopic scale [24]. When the difference in orientations between adjacent grains is large, grains with a soft orientation are severely deformed and form a “valley” morphology, while grains with a hard orientation are not easily deformed and form a “peak” morphology. It can be seen that the difference in Schmid factors of the adjacent grains connects the externally applied stresses with the critical shear stresses to a specific slip system, which ultimately causes the changes in thickness and free surface irregularities. The surface morphology of microtensile deformed specimens is illustrated in Figure 6. On the surface of the specimen, “peak” and “valley” morphologies can be observed. On the mesoscopic scale, a polycrystal is considered to be a collection of many individual grains with different orientations. Therefore, plastic deformation does not mean all grains slide simultaneously. If each grain is deformed individually, voids and overlaps will appear at the grain boundaries, which is inconsistent with the global continuity. Single grain may slip and rotate while the grains are hindered by the surrounding grains. Therefore, the stresses are transferred to the surrounding grains, resulting in the deformation of the surrounding grains to achieve the continuity of plastic deformation. When only a few grains exist in the thickness of the thin sheet specimen, the deformation of grains is restricted by the free surface. The number of slip systems activated to adapt to local plastic deformation is reduced. The deformation tends to be localized at the grain boundary, and additional shear displacement is required for grain rotation to maintain the continuity of the deformation on the grain boundary. The roughening of the free surface generated during the deformation of the specimen will cause the strain localization in the subsequent deformation, resulting in a decrease in the fracture strain. Therefore, the roughening of the free surface in the mesoscale plastic deformation will affect the deformation behavior, ductility, and fracture morphology of the material, and then affect the dimensional accuracy and surface quality of the micro-components. As the plastic deformation increased from ε = 0.1 to ε = 0.4, the surface roughness of the specimen increased significantly, which indicated that the plastic deformation increases the surface roughness. The surface roughness of the specimens decreased with the increasing volume fraction of the δ phase, which indicated that the presence of the δ phase reduced the roughness of the free surface. This is mainly because the δ phase restricted the rotation of the grains in the surface layer during microtensile deformation, i.e., it affected the evolution of grain orientation during plastic deformation. Thus, it limited the deformation of adjacent grains with a soft orientation.

### 3.4. Local Strain Evolution Behavior

**Figure 7:**
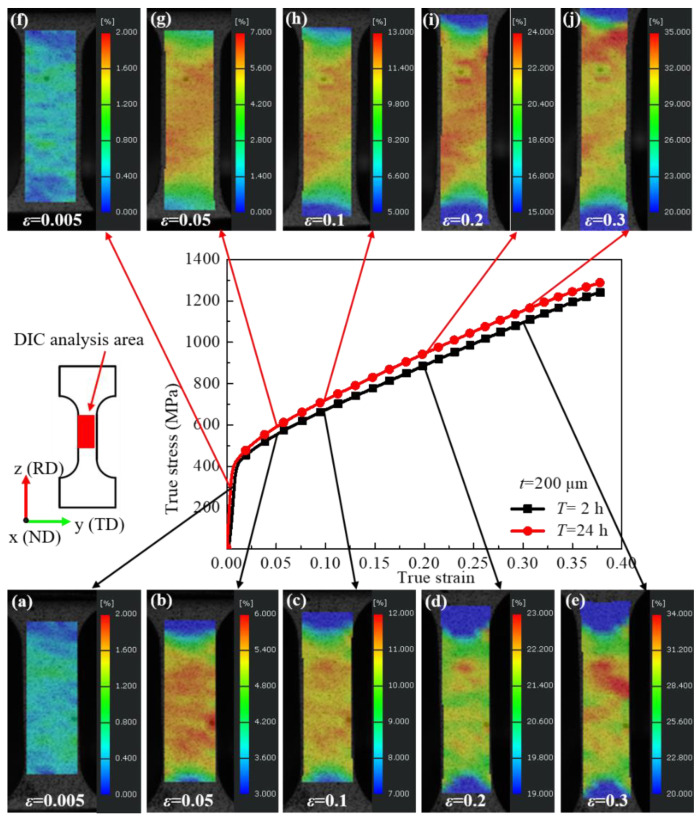
Strain evolution behavior of the alloy during the tensile process. (**a**–**e**) Different tensile strain stage of the alloy with the aging time of 2 h; (**f**–**j**) different tensile strain stage of the alloy with the aging time of 24 h.

The DIC technique enables the characterization of the deformation behavior by calculating the strain field during deformation. The results of strain evolution during microtensile characterized by DIC are shown in Figure 7. It can be seen that the specimens aged for 2 h already showed significant inhomogeneous deformation in the elastic deformation stage. When the true strains were ε = 0.05, ε = 0.1, ε = 0.2, and ε = 0.3, the maximum local strain reached ε_max_ = 0.060, ε_max_ = 0.115, ε_max_ = 0.227, and ε_max_ = 0.333, respectively. It was found that the degree of inhomogeneous plastic deformation gradually increased after yield, and the strain concentrations appeared in several local regions. The maximum values of local strains reached ε_max_ = 0.065, ε_max_ = 0.122, ε_max_ = 0.240, and ε_max_ = 0.348 for the specimens aged for 24 h with true strains of ε = 0.05, ε = 0.1, ε = 0.2, and ε = 0.3, respectively. It can be found that the strain localization degree of specimens aged for 24 h is slightly higher than that of specimens aged for 2 h. The strain evolution behavior is closely related to the morphology and distribution of the δ phase during microtensile deformation. The morphologies of the δ phase were spherical, rod-like, and needle-like in different grains, and the particle size and orientation distribution of the δ phase were also different. During the microtensile deformation, the dislocation motion was hindered by the δ phase, and the dislocation motion was blocked and accumulated near the δ phase, which was unfavorable to the plastic deformation behavior and detrimental to the ductility of GH4169 nickel-based superalloy.

### 3.5. Fracture Morphology and Fracture Mechanism

**Figure 8:**
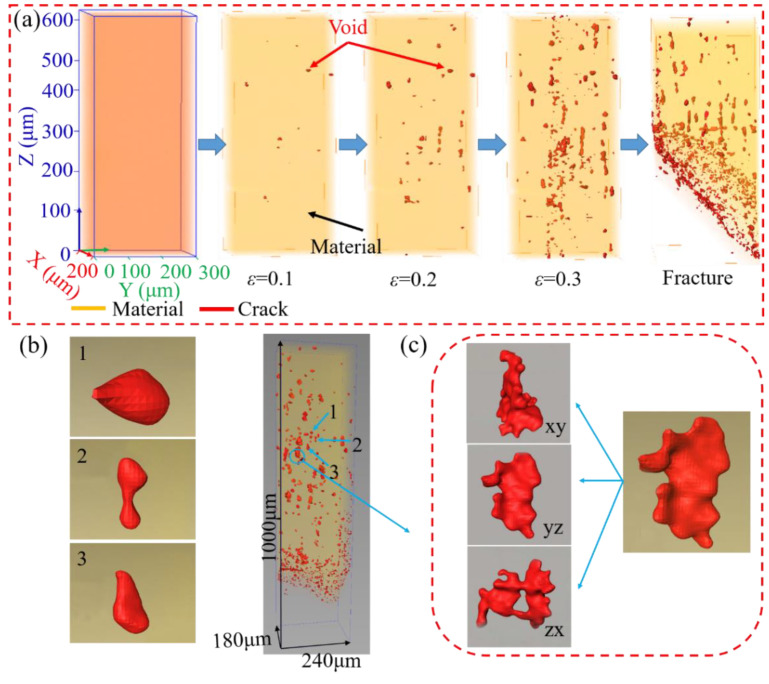
3D reconstruction results of voids for the alloy with δ phase. (**a**) Distribution of voids at various true strains; (**b**,**c**) morphologies of the voids.

The δ phase plays an essential role in the mechanical properties and fracture behavior. A large number of studies have been carried out on the effect of δ on the mechanical properties of the superalloy on the macroscopic scale. However, the effect of δ on the voids’ initiation and propagation during plastic deformation has only been qualitatively described based on the two-dimensional (2D) fracture morphology. No research has been carried out on the three-dimensional (3D) characterization. In order to reveal the influence of the δ phase on the void initiation and propagation during microtensile deformation, the 3D reconstruction results of specimens after in situ microtensile are shown in Figure 8. It can be found that the void size and volume fraction gradually increased with the increase of plastic strain, as shown in Figure 8a. Additionally, some small voids expanded into large voids with complex morphology during microtensile deformation, as shown in Figure 8b,c, which is closely related to the fact that the δ phase provides a channel for the expansion of voids and cracks.

**Figure 9:**
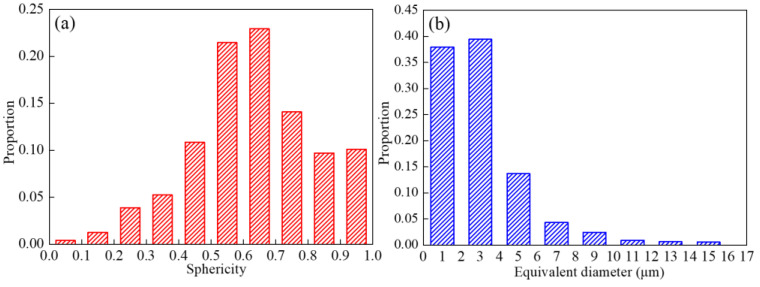
Void characteristics of the fractured specimens. (**a**) Sphericity distribution; (**b**) ED distribution.

The results of the quantitative statistical analysis of the sphericity and equivalent diameter of the voids are shown in Figure 9. The sphericity of the voids mainly varies between 0.5 and 0.8, which indicates that the shapes of the voids evolve into ellipsoids during the microtensile deformation. The equivalent diameters of the voids mainly vary between 1 μm and 6 μm, and some complex shaped voids with larger equivalent diameters occur.

**Figure 10:**
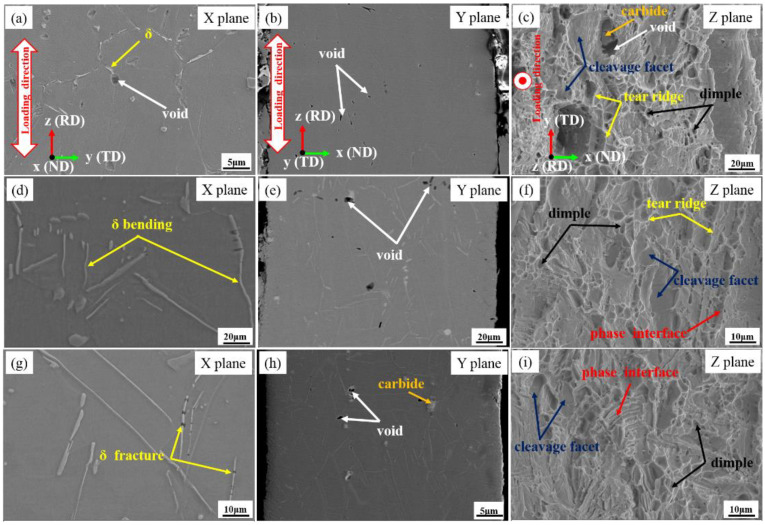
Fracture morphologies from the X, Y, and Z planes for the specimen under the condition of various aging times. (**a**–**c**) 2 h; (**d**–**f**) 12 h; (**g**–**i**) 24 h.

The fracture morphologies in the X, Y, and Z planes of the specimens with different aging times are shown in Figure 10. Through observing fracture morphologies in the X and Y planes, it can be found that the needle-like δ phases at and near the fracture were pulled off into short rods and some δ phases were bent after the uniaxial microtensile deformation. In contrast, the spherical δ phases with smaller sizes did not fracture. Therefore, the fracture behavior of nickel-based superalloy is closely related to the morphology and distribution of the δ phase. In our previous study, the fracture mechanism of the δ phase was revealed and the fracture model of the δ phase was established [25]. When the angle between the loading direction and the long-axis direction of the δ phase is slight, the critical dislocation density required for fracture is much lower than that required for a larger angle. Therefore, the fracture of the δ phase is more likely to occur when the angle between the long-axis direction of the δ phase and the loading axis is slight. For the spherical δ phase, the critical dislocation density required for fracture is much higher than that of the rod-like and needle-like δ phase because its aspect ratio is much smaller. Therefore, the fracture of the spherical δ phase is much more difficult than that of the rod-like and needle-like δ phase. Thus, the reduction in the volume fraction of the rod-like and needle-like δ phase is conducive to service performance. By observing fracture morphologies in the Z plane, it can be found that a large number of equiaxial dimples exist in the fracture when the volume fraction of the δ phase is small (Figure 10c). Some carbide fragments exist inside some voids. The presence of large voids is attributed to the carbide particles having a brittle nature and facilitate the crack nucleation sites [26,27]. The tear ridges and cleavage facets also occur at the fracture surface. As shown in Figure 10f, the density and depth of deep dimples is observed to be decreased. As shown in Figure 10i, the density of dimples is further reduced and the dimples only appear in the local area. Additionally, the area of cleavage facets increases while the density and the depth of dimples are reduced. The dimples observed at the fracture surface are associated with the plastic deformation. This indicates that the ductility of the alloy decreases with the increasing volume fraction of the δ phase. Many voids with larger sizes appeared on the fracture surface and the fracture became not flat. Additionally, a “bar” groove morphology was found, which is closely related to the particle size and distribution of the δ phase.

The deformation of the surface layer grains mainly depends on their own properties and orientation, because the deformation behavior of surface layer grains is similar to that of the single crystal during mesoscopic-scale plastic deformation. As each grain in the surface layer has a different orientation, the rotation of the grains will lead to the surface roughing, which further results in the strain localization in the thickness direction of specimens. The δ phase impedes the motion of dislocations during the plastic deformation, which contributes to dislocation piling up around the δ phase. The impediment mechanism to the dislocation motion is the same owing to the appearance of the δ phase in both the surface layer grains and the internal grains. The δ phase can limit the rotation of surface layer grains and reduce the surface roughening. Additionally, the strength of surface layer grains can be improved, and the deformation incompatibility between surface layer grains and internal grains can be weakened by the δ phase. However, the interface between the δ phase and matrix γ phase is incoherent, resulting in weak interfacial bonding strength. The intense stress concentration will be generated near the phase interface while the external force is applied. The δ phase can debond from the matrix or fracture when the stress concentration caused by dislocation piles up, reaching the phase interface strength or the fracture strength of the δ phase. The voids will nucleate at the above fracture locations, which are often located within the crystal. The voids will grow and aggregate as plastic deformation continues. The stress required to emit dislocations on the surface of the void decreases with the increase in the radius of the void, indicating that the existence of the void advances the failure time. When voids meet with each other, they will join together and form microcracks. Additionally, under the action of the applied stress, the rod-like and needle-like δ phase provides a channel for microcrack expansion, i.e., a “bar” groove morphology on the fracture surface. The cracks gradually expand to the surrounding area until the cracks converge and eventually lead to the fracture of the specimen.

## 4. Conclusions

In this study, microtensile experiments were carried out for specimens with various morphologies and volume fractions of δ phases. The effect of the δ phase on the grain orientation, surface roughening, and strain localization was investigated. The microtensile fracture mechanism was revealed. The main conclusions are as follows.

The average KAM values of the fracture specimens are higher than those of the heat-treated specimens. The surface roughness of the specimens increases significantly as the plastic deformation increases from ε = 0.1 to ε = 0.4. The surface roughness decreases with the increase in δ phase volume fraction, which means that the presence of the δ phase reduces the degree of roughening.

When the true strains were ε = 0.05, ε = 0.1, ε = 0.2, and ε = 0.3, the maximum local strain of specimens aged for 2 h reached ε_max_ = 0.060, ε_max_ = 0.115, ε_max_ = 0.227, and ε_max_ = 0.333, respectively, while the maximum values of local strains reached ε_max_ = 0.065, ε_max_ = 0.122, ε_max_ = 0.240, and ε_max_ = 0.348, respectively, for the specimens aged for 24 h. The increasing particle size and volume fraction of the δ phase intensified the degree of strain localization.

The fracture of the spherical δ phase is much more difficult than that of the rod-like and needle-like δ phase. Under the action of the applied stress, the rod-like and needle-like δ phase provided a channel for microcrack expansion, and the cracks gradually expanded to the surrounding area until the cracks converged and eventually led to the fracture of the specimen.

## Figures and Tables

**Table 1:** Chemical composition of GH4169 nickel-based superalloy (wt%).

Element	Ni	Cr	Nb	Mo	Al	Ti	C	Co	Fe
ASTM	50–55	17–21	4.75–5.50	2.8–3.3	0.2–0.8	0.65–1.15	<0.08	<1.0	Bal.
as-received	52.80	18.73	5.24	3.02	0.52	0.95	0.03	0.03	Bal.

## Data Availability

Data sharing is not applicable for this article.
